# Grade III Splenic Laceration After a Ground-Level Fall in a Pediatric Patient: The Need for Return-to-Play/Activities Protocols for Individuals With Splenic Injury or Splenomegaly

**DOI:** 10.7759/cureus.42610

**Published:** 2023-07-28

**Authors:** Andrew Baird, Christopher Pun, Asfawossen Asfaw

**Affiliations:** 1 Family Medicine, Grand Strand Medical Center, Myrtle Beach, USA; 2 School of Medicine, Edward via College of Osteopathic Medicine - Carolinas Campus, Spartanburg, USA; 3 Pediatric Critical Care, Grand Strand Medical Center, Myrtle Beach, USA

**Keywords:** return-to-play protocol, splenic laceration, splenic injury

## Abstract

Splenic injury is a potentially fatal injury if left undetected or untreated. Although most splenic injuries result from a traumatic event, it is important to consider if one's history (past or present) increases their risk for splenic injury (i.e., splenomegaly). We present a case regarding a school-age child who presented to the Emergency Department (ED) with abdominal pain following a ground-level fall onto a carpeted stair step. Prior to this injury, the patient had cold-like symptoms for 3 months that were treated solely with supportive care by their pediatrician(s). A transferring hospital’s abdominal CT imaging revealed a grade III splenic laceration. The patient was monitored in the pediatric intensive care unit (PICU) by way of serial abdominal examinations, vitals, and labs. When the patient was cleared for discharge, it was recommended to refrain from strenuous activity for 1-2 months due to the risk of repeat splenic injury. Post-discharge, the patient's Epstein-Barr virus (EBV) serology returned and was consistent with a past infection which was an inconclusive finding. Although trauma is most commonly the culprit of splenic injuries, it is important to keep differentials broad when considering causes of splenomegaly as this may allow healthcare providers to potentially prevent injury/provide appropriate management post-injury and guide return-to-play recommendations.

## Introduction

It is well established that the spleen is the most frequently injured organ in association with abdominal trauma. Splenic injury can be classified into traumatic and non-traumatic - traumatic being more prevalent than non-traumatic [1]. Traumatic splenic injuries most commonly occur via motor vehicle accidents or direct abdominal blows which commonly occur in sports, while non-traumatic causes included neoplasm, infectious, inflammatory disease, medication and medical treatment, mechanical causes, and idiopathic [2]. Non-traumatic causes can often be conspicuous, making a patient’s past medical history and review of symptoms important to help reveal any predisposing factors for splenic injuries (such as infectious mononucleosis (IM) and hematological disorders) as these cause splenic enlargement and capsule thinning, which lead to splenic fragility [3].

## Case presentation

A 6-year-old, previously healthy boy, was transferred from another hospital to a level II Pediatric Trauma Center after CT abdomen revealed splenomegaly and grade III splenic laceration. Per the patient’s mother, who was at the bedside, the patient had an unwitnessed ground-level fall at home. His mother noted that the patient was running in the playroom when he fell forward onto his abdomen, and she believed he fell into the carpeted stairstep. She reported that the patient immediately complained of abdominal pain and increased work of breathing. As a result, the parents then took him to the ED at another hospital. Of note, the mother stated that the patient had lingering cough/cold symptoms for approximately three months which led to multiple pediatrician visits and resulted in supportive care treatments only.

Upon arrival at the ED from the transferring hospital, the patient was immediately seen by the trauma team. The patient’s vital signs were as follows - blood pressure of 103/62 mmHg, heart rate of 109 beats/min, respiratory rate of 29 breaths/min, and temperature of 97.8F. Physical exam was notable for tenderness to palpation over the periumbilical area, but the abdomen was soft, non-tender, and without guarding. A bedside focused assessment with sonography in trauma (FAST)** **exam showed no free fluid present. The patient was admitted to the PICU for further observation and monitoring.

**Figure 1:**
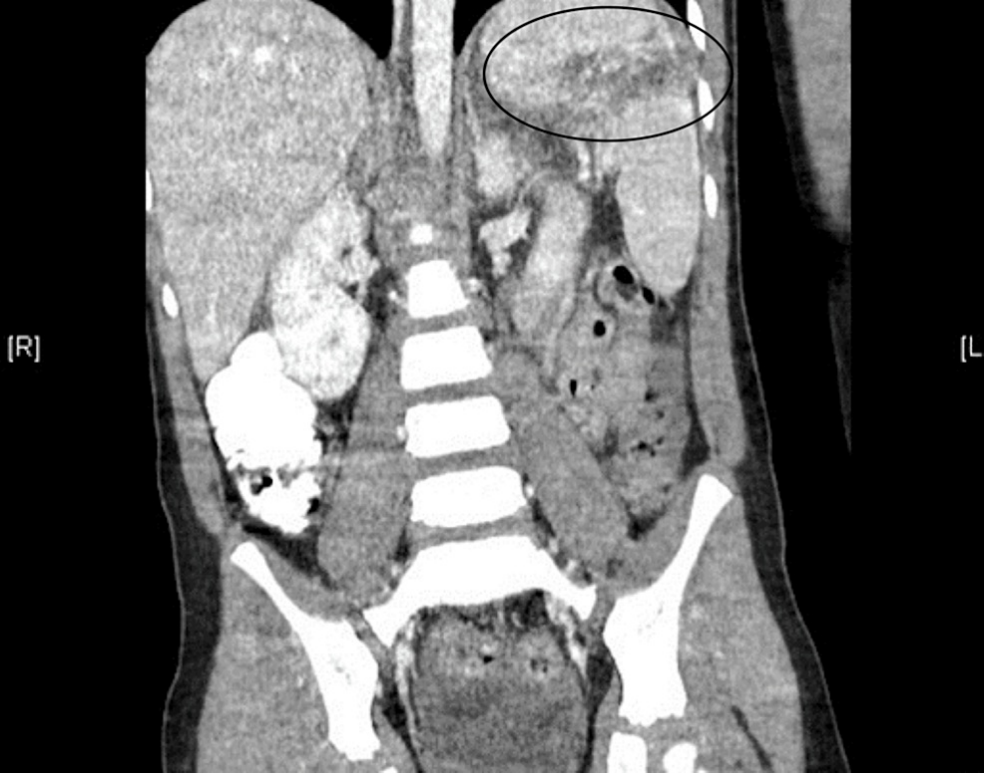
CT abdomen (coronal) consistent with splenic laceration

**Figure 2:**
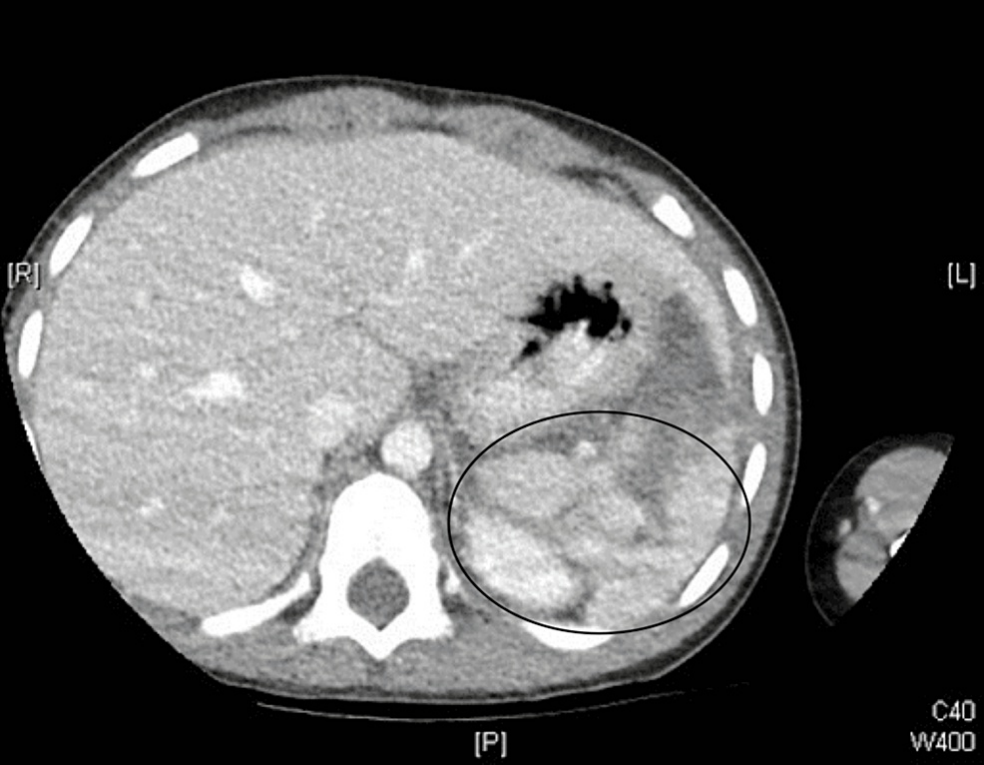
CT abdomen (axial) consistent with splenic laceration

No additional imaging was pursued, as access to the CT abdomen from the transferring hospital was available (Figure 1-2). The patient's initial laboratory workup consisted of a complete blood count (CBC), comprehensive metabolic panel (CMP), monospot assay, and Epstein-Barr virus (EBV) serology. CBC was significant for a hemoglobin of 10.8 (normal: 11.2-14.5 g/dL), hematocrit of 32.4 (normal: 35-44%), platelet count of 401 (normal: 150-450 x10^9/L), and white blood cell count of 12 (normal: 4.8-10.8 x10^3/uL). CMP was grossly unremarkable and essentially within normal limits.

Given the patient's hemodynamic status, the patient was managed supportively with maintenance IV fluids, multimodal pain control, serial CBCs every 6 hours, and serial abdominal exams. The patient's serial abdominal exams remained unchanged, diet progressed as tolerated, and the patient's hemoglobin/platelets remained stable/trended upward. The patient was then discharged by the trauma team with the following discharge instructions - “Given patient is at high risk for repeat splenic injury and rupture, patient should refrain from strenuous activity and contact sports for minimum 1 month, preferably 2 months. Patient should follow up with a pediatrician for close follow up and creation of a follow up plan.”

Results of the monospot assay and EBV serology came back a few days after discharge. The results are as follows: monospot assay - negative; EBV DNA - negative, EBV Capsid Ag IgG Ab 132 - High, EBV Capsid Ag IgM Ab <36 - normal, and EBV nuclear Ag Ab 71.8 - High. These results are consistent with a past/prior EBV infection (Table 1).

**Table 1:** Interpretation of EBV Serology Profiles VCA: Viral Capsid Antigen, EBNA: Epstein-Barr virus Nuclear Antigen; IgM: immunoglobulin M; IgG: immunoglobulin G; Ab: anitbodies

Interpretation	VCA IgM	VCA IgG	EBNA Ab
Acute infection	+	-	-
Past/Previous infection	-	+	+
Acute or Past infection	-	+	-

## Discussion

Splenomegaly and consequent risk of rupture is the primary concern when determining an athlete’s restriction from activity and return to contact sports. Although the majority of splenic injuries result from a traumatic blow, it is important to consider non-traumatic causes of injury - neoplasm, infectious, inflammatory disease, medication, medical treatment (iatrogenic), and idiopathic [2].

Even though our patient's splenic injury was likely due to the known abdominal trauma from a ground-level fall, one could consider if the patient was predisposed or at an increased risk for splenic injury. The patient's mother reported a history of lingering cough/cold symptoms that failed to improve with standard supportive care measures. Although the patient's serology was consistent with previous EBV infection, it cannot clearly be connected as a contributor to this specific case. Nonetheless, EBV is known to cause splenomegaly, and before the age of 10, primary EBV infection is usually asymptomatic or only present with a variety of minor symptoms consistent with upper respiratory infections, otitis media, or abdominal complaints [4]. EBV is associated with multiple disease processes, but it is mostly recognized as the main infectious agent for infectious mononucleosis (IM) [5]. Not only is the pediatric population overlooked due to the lack of typical IM symptoms, essentially all heterophile/monospot assays may be falsely negative [4,5]. Given that EBV-associated IM has been shown to vary in presentation across different age groups, remaining vigilant and aware of atypical symptoms when working up patients is essential for diagnosis and ultimately reducing the risk of splenic rupture. Although our patient's EBV serology showed evidence of a past infection as opposed to an acute/primary infection, the presumptive past EBV infection could have put the patient at a theoretical increased risk. Therefore, nonspecific presentations increase the odds of a missed diagnosis with a subsequent increased risk of splenic rupture as a result.

Currently, there is a lack of consensus for when athletes, who have been diagnosed with IM, are safe to return to contact sports. At the bare minimum, they should be asymptomatic from fever, pharyngitis, and fatigue prior to considering resuming light activity [6-8]. Determining optimal return to activities and sports with increased risk of abdominal trauma is the more difficult task and may require more individualized discussion. Recommendations for activity restriction have ranged from a minimum of three weeks to as long as three months due to the variable disease course and the risk of a delayed rupture [9,10]. Sergent et al. recommend that patients avoid contact sports for a minimum of four weeks or until the resolution of splenomegaly [9]. Sylvester et al. consider extending the guideline to a minimum of 31 days following symptom onset of IM due to finding a significant portion of patients with splenic ruptures occurring between 21-31 days after onset [5]. Thus, the risk of splenic rupture may be highest during the first 3-4 weeks. Additionally, in a study of 20 patients with IM, peak splenic size was typically observed to be during the first two weeks after symptom onset using ultrasound (US), and most cases of splenomegaly resolved within 4-6 weeks after symptom onset [3]. When considering the use of ultrasonography (US) to monitor the resolution of splenomegaly in patients with IM, it seems that its use is physician and athlete dependent. O’Connor et al. studied the usage of abdominal US one month following the diagnosis of IM as a possible tool to guide return-to-play recommendations in a group of 19 athletes [11]. At the one-month follow-up, 16 athletes had a spleen size < 12 cm and were allowed to return to normal activity and contact sports. The remaining patients with spleens >12 cm were recommended to continue restriction of activity and follow up for repeat US at month two [11]. This allowed for patients to return to play sooner than other guidelines may have advised and may have also prevented injury in those with abnormal spleen sizes at the one-month mark. Therefore, for patients who may be more serious athletes that would like to resume competitive sports as soon as possible or athletes with high-grade splenic injury and persistent symptoms, abdominal US may be a beneficial tool to allow for individualized informed decision-making. For patients already presenting with blunt splenic injury or rupture, there is more data supporting guidelines for return to play. This is based on the grading system by the American Association for the Surgery of Trauma, which classifies blunt splenic injuries from I to V with contrast-enhanced CT imaging [12]. The Updated American Pediatric Surgical Association (APSA) Guidelines for the management of blunt liver and spleen injuries recommend restricting activities based on the injury grade plus two weeks [13]. Although a shorter duration of activity restriction may be safe, there is inadequate data supporting it. Additionally, under the APSA guidelines, repeat imaging following blunt splenic injury is not indicated in asymptomatic patients, but may be considered in those with persisting symptoms and high-grade injury [13].

## Conclusions

While there is data to support activity restriction guidelines in athletes with blunt splenic injury, there is insufficient data for an optimal return-to-play protocol in those suffering from splenic injury/laceration. Guidelines for resuming activity remain controversial and conflicting, with recommendations varying from weeks to months. At the bare minimum, an individual should be monitored closely and decisions regarding spleen monitoring should be shared between the individual and their respective provider(s) when considering return to play or return to activities. Ultimately, more studies and data regarding splenic healing are needed before any safe and general guidelines can be recommended.
